# Supplementary material for: Content-rich biological network constructed by mining PubMed abstracts
Source: BMC Bioinformatics. 2004 Oct 8;5:147. doi: 10.1186/1471-2105-5-147 (PMC528731; doi:10.1186/1471-2105-5-147)
Supplement: Additional File 2 — The original results of the above study (non-essential files are deleted to keep the file size under the limit set by BMC bioinformatics). [file 1471-2105-5-147-S2.bz2 › chilibotAdditionalFile2/dip20/234ID8006584E758/html/IL2RB_IL2RA.html]

 


 **IL2RB** and **IL2RA** 
  
Found 1183 abstracts in PubMed, retrieved 20.  
 

 What does Google say? 
 PDF only 
| .edu only 

---

**Interactive relationship** (e.g. stimulation, inhibition, etc)

**Non-interactive relationship** (e.g. studied together, co-existance, homology, etc.)

- Cell surface expression of CD25  [ **IL2RA** ] , CD122  [ **IL2RB** ] , CD132, and CD69 were also determined and no statistical differences were found between both groups.  Ref: 12635575 Allergy Asthma Proc< MedlineTA>Allergy Asthma Proc,
- Cells stained with Annexin V Anx and antibodies to CD56, CD3, CD95, CD25  [ **IL2RA** ] , CD122  [ **IL2RB** ]  or CD132 were examined by flow cytometry.  Ref: 12594840 Eur J Immunol, 2003
- CONCLUSION The monoclonal antibody against IL 2 receptor  [ **IL2RB** ]  alpha chain CD 25  [ **IL2RA** ]  has a marked effect upon severe steroid refractory GVHD.  Ref: 12812665 Zhonghua Yi Xue Za Zhi, 2003
- Most of the surface membrane antigens CD5, CD11c, CD19, CD20, CD21, CD22, CD23, CD25  [ **IL2RA** ] , CD40, CD45, VD79b, CD80, CD95, CD122  [ **IL2RB** ] , CD124, CD126, CD130, CD154, IgM, and IgD showed a similar expression pattern in both UM CLL and M CLL patients.  Ref: 12631259 Eur J Haematol, 2003
- We observed that eosinophils from most patients with hypereosinophilia express the alpha chain of the IL 2 receptor  [ **IL2RB** ]  CD25  [ **IL2RA** ] ,.  Ref: 12672048 Eur J Immunol, 2003
- Parameters of cellular and humoral immunity differential blood count, T cell markers CD2, CD3, CD4, and CD8, B cell markers CD19 and CD20, monocyte markers CD13 and CD14, NK cell marker CD16, activation markers CD25  [ **IL2RA** ] , CD26, CD69 and HLA DR, and cytokines IL 1 receptor antagonist IL 1RA, IL 2, soluble IL 2 receptor  [ **IL2RB** ]  sIL 2R, IL 6, IL 10, and TGFbeta were measured in peripheral venous blood.  Ref: 12779014 Folia Biol Praha , 2003
- The inhibitory effect on human T cells was assessed and demonstrated at the level of proliferation DNA synthesis, IL 2 secretion, and IL 2 receptor  [ **IL2RB** ]  CD25  [ **IL2RA** ]  upregulation.  Ref: 12835898 Inflamm Res, 2003
- Most investigated Treg cells are CD4 pos cells expressing the alpha chain of the IL 2 receptor  [ **IL2RB** ]  CD25  [ **IL2RA** ] .  Ref: 12848995 Autoimmun Rev, 2002
- BACKGROUNDTo define whether immunosuppressive agents that block the interleukin IL 2 pathway could prevent activation induced cell death of activated T cells in the graft, we measured expression of IL 2, IL 2 receptor  [ **IL2RB** ]  alpha chain CD25  [ **IL2RA** ] , IL 15, Fas, and Fas ligand by real time reverse transcription polymerase chain reaction in cardiac allografts.METHODSWe characterized the phenotype of the infiltrating cells CD3, CD68, CD25  [ **IL2RA** ]  by immunohistochemistry.  Ref: 12777860 Transplantation, 2003
- When we compared mean nocturnal levels, CD16 and CD25  [ **IL2RA** ]  T and B activated lymphocytes with expression of the a chain of IL 2 receptor  [ **IL2RB** ]  levels were higher, while CD8, CD8bright, CD20 total B cells, TcRd1 epitope of the constant domain of d chain of T cell receptor 1 and dTcS1 epitope of the variable domain of d chain of T cell receptor1 levels were lower in cancer patients.  Ref: 12797908 Int J Immunopathol Pharmacol< MedlineTA>Int J Immunopathol Pharmacol, 2003
- We investigated a possible relation between prolactin receptor PRL R or IL 2 receptor  [ **IL2RB** ]  alpha IL 2Ralpha, CD25  [ **IL2RA** ]  expression on circulating T lymphocytes and their apoptosis in patients with breast cancer.  Ref: 12698200 Br J Cancer, 2003
- Activation markers CD25  [ **IL2RA** ] , CD122  [ **IL2RB** ] , CD69 and CD80 were also present on fetal T cells.  Ref: 12880639 Dev Comp Immunol, 2003
- We found a defective T cell response in malnourished children, as indicated by diminished levels of circulating total CD3, helper CD4, IL 2 receptor  [ **IL2RB** ]  bearing CD4 CD25  [ **IL2RA** ]  and memory helper T cell responses CD4 CD45RO in keeping with the decreased specific IgE levels against Ascaris lumbricoides.  Ref: 12848201 J Trop Pediatr, 2003
